# Alterations of Plasma Lipids in Adult Women With Major Depressive Disorder and Bipolar Depression

**DOI:** 10.3389/fpsyt.2022.927817

**Published:** 2022-07-18

**Authors:** Ting Zhang, Lin Guo, Rui Li, Fei Wang, Wen-mao Yang, Jia-bin Yang, Zhi-quan Cui, Cui-hong Zhou, Yi-huan Chen, Huan Yu, Zheng-wu Peng, Qing-rong Tan

**Affiliations:** ^1^Department of Psychiatry, Chang’an Hospital, Xi’an, China; ^2^Department of Psychiatry, Xijing Hospital, Air Force Medical University, Xi’an, China

**Keywords:** lipidomics, women, depression, bipolar depression, plasma lipid

## Abstract

Lipidomics has been established as a potential tool for the investigation of mental diseases. However, the composition analysis and the comparison of the peripheral lipids regarding adult women with major depressive depression (MDD) or bipolar depression (BPD) has been poorly addressed. In the present study, age-matched female individuals with MDD (*n* = 28), BPD (*n* = 22) and healthy controls (HC, *n* = 25) were enrolled. Clinical symptoms were assessed and the plasma samples were analyzed by comprehensive lipid profiling based on liquid chromatography-mass spectrometry (LC/MS). We found that the composition of lipids was remarkably changed in the patients with MDD and BPD when compared to HC or compared to each other. Moreover, we identified diagnostic potential biomarkers comprising 20 lipids that can distinguish MDD from HC (area under the curve, AUC = 0.897) and 8 lipids that can distinguish BPD from HC (AUC = 0.784), as well as 13 lipids were identified to distinguish MDD from BPD with moderate reliability (AUC = 0.860). This study provides further understanding of abnormal lipid metabolism in adult women with MDD and BPD and may develop lipid classifiers able to effectively discriminate MDD from BPD and HC.

## Introduction

Major depressive disorder (MDD) and bipolar disorder (BD) are highly prevalent affective disorders, leading to increased adverse sequelae of other common comorbid medical conditions and suicide risk and a heavy social burden (1–3). The lifetime prevalence of MDD is evaluated to be around 20% and is predicted to be the leading cause of global disability by the year 2030 according to the World Health Organization (4, 5), whereas the lifetime prevalence range of BD is from 0.5 to 2.4% worldwide (6, 7). Although hypomania and mania are the most recognizable characteristics, patients with BD were hypomanic or manic less than 10% of the time, and depression is its most frequent clinical presentation (8, 9). Meanwhile, the overlapping symptomology between MDD and bipolar depression (BPD) is very common (10, 11). Although the pharmacotherapeutic strategies in these two disorders are substantially different (12, 13), patients with BPD are often initially misdiagnosed with depression (14). In addition, diagnosis may change from depression to BPD over the long-term disease progression (15), which may lead to inappropriate treatment and greater healthcare costs (16). Meanwhile, the benefit of drug treatment is still unsatisfactory for patients even with a definite diagnosis. For instance, the summary effect sizes of antidepressants are mostly modest (17) and approximately 30% of MDD do not respond to standard pharmacological therapies (18). On the other hand, the efficiency of drug treatments for BD is about only 40–60% and usually causes side effects (19, 20). As a result, the underlying molecular basis and correct diagnosis for MDD and BPD remain largely concerned (21, 22).

Women are nearly twice as likely as men to develop depression during their lifetime (23, 24) and women with MDD also report greater illness severity and increased suicide attempts than men (25–27). On the other hand, although there is no gender difference in the incidence rate of BD, women are more likely to have depressive episodes and bring more burden of disease than men (28–30) and are at high risk of having a serious episode of illness during childbirth and pregnancy (31, 32). However, the objective biomarkers that can distinguish women with MDD from BD have not been identified. Thus, clarifying the pathological features of MDD and BD in women might be able to provide a basis for the development of new diagnostic methods and treatment strategies.

The use of peripheral blood as the sample source for identifying diagnostic, prognostic, and predictive biomarkers for MDD and BD has been already reported (33–35). Correspondingly, lipids from peripheral blood already represent a target for preventive medicine for MDD and BD (36). Recently, lipidomics analyses have also demonstrated the role of serum lipid abnormalities in BD and MDD (37, 38). For example, targeted lipidomics reveals that high plasma ceramides in patients with MDD and BD are indicative of a high metabolic burden (39). Although previous plasma lipidomics analyses have revealed the characteristic lipid molecules of antenatal depression and female bipolar disorder (40, 41), thus far, there are only limited understandings of the role of lipids in the pathophysiology and the direct comparison of lipid compositions between adult MDD and BPD in women has not been reported. Such investigations might shed a light on the identifications of peripheral biomarkers for these two disorders.

Considering the above, the present study performed a case-control study of lipidomic analysis of plasma samples (*n* = 75) from age-matched female individuals with MDD (*n* = 28) and BPD (*n* = 22) compared with healthy controls (HC, *n* = 25) by using liquid chromatography-mass spectrometry (LC/MS). Next, we identified female MDD- and BPD-related lipidomic signatures compared to HC by orthogonal partial least-squares discrimination analysis (OPLS-DA) as well as discriminative lipids panels that could distinguish MDD, BPD, and HC by random forest and receiver operating characteristic (ROC) analysis. Finally, correlation analysis based on the relative abundance of altered lipids and clinical symptom scores was performed to explore the potential lipid function.

## Materials and Methods

### Subjects and Plasma Sampling

In total, 28 female patients who met the DSM-5 criteria for MDD (24–46 years of age) or 22 female patients for BPD (26–58 years of age) were recruited from the Department of Psychiatry at Chang’an Hospital, Xi’an, China, along with 25 healthy female (25–53 years of age) individuals, all of which were Han nationality and underwent a physical examination. The Mini-International Neuropsychiatric Interview was used to screen for pre-existing psychiatric disorders. Diagnosis of MDD and BPD was performed by two senior psychiatrists based on a Structured Clinical Interview for Diagnostic and Statistical Manual of Mental Disorders, 5th Edition’s (DSM-5) criteria. All the patients with BPD were recruited with a current depressive episode. Meanwhile, psychiatrists who had attended a training session on how to administer the tests before the start of the study and were blinded to the clinical status of the participants evaluated the clinical symptoms by the Hamilton Anxiety Scale (HAM-A), Hamilton Depression Rating Scale (HAM-D) and Positive and Negative Syndrome Scale (PANSS) scores. The exclusion criteria include: patients with inflammatory bowel disease, Crohn’s disease or other diseases of the digestive system, patients with other organic diseases; hypertension; obesity, defined as a body-mass index (BMI) ≥ 28.0; pregnancy, lactation or menstrual period; severely unbalanced diet, such as high-fat diet or long-term vegetarians; the presence of other mental disorders according to the DSM-5 criteria.

Blood samples were collected in anticoagulant tubes and centrifuged at 1,600 rpm for 15 min, and then aliquoted into sterile cryopreservation tubes and stored in liquid nitrogen until analysis. Blood samples were collected between 8 and 10 a.m. with all individuals under fasting conditions.

### Lipidomics Analyses

The lipidomics analyses were supported by Shanghai Applied Protein Technology Co., Ltd., which were performed as previously described (42, 43). Briefly, plasma (100 μL) was spiked with internal lipid standards (SPLASH^®^ LIPIDOMIX^®^ Mass Spec Standard, methanol solution, AVANTI, 330707-1EA, Merck, United States) and homogenized with water and methanol, then 800 μL of methyl tert-butyl ether (MTBE, Thermo Fisher, United States) was added. After centrifuging, the supernatant was separated for analysis. For LC-MS, samples were separated by a UHPLC Nexera LC-30A ultra-high-performance liquid chromatography system with a C18 column (ACQUITY UPLC CSH C18, 1.7 μm, 2.1 mm × 100 mm, Waters, United States). The lipid extracts were re-dissolved in 200 μL 90% isopropanol/acetonitrile (Thermo Fisher Scientific, United States), centrifuged at 14,000 *g* for 15 min, and finally, 3 μL of the sample was injected. Mass spectra were acquired by Q-Exactive Plus (Thermo Fisher Scientific, United States) in positive and negative modes, respectively. Electrospray ionization (ESI) parameters were optimized and preset for all measurements as follows: Source temperature, 300°C; capillary temperature, 350°C, the ion spray voltage was set at 3000V, S-Lens RF Level was set at 50% and the scan range of the instruments was set at m/z 200–1,800. Identification of lipids was performed by LipidSearch™ software (Thermo Fisher Scientific, United States), which contains more than 30 lipid classes and more than 1,500,000 fragment ions in the database. Single-point internal standard calibrations were used to estimate absolute concentrations for unique lipids identified by accurate mass, MS/MS spectral match, and retention times (44).

### Statistical Analyses

Differences in continuous variables were assessed with the one-way Analysis of Variance (ANOVA) or Kruskal Wallis test. The data of lipids was further converted through log10 and standardized by Pareto scaling. Then the data quality was evaluated by the partial least squares discriminant analysis (PLS-DA) and the orthogonal partial least squares discriminant analysis (OPLS-DA) method was applied to remove irrelevant variables. The quality of the OPLS-DA was validated by the permutation test.

In addition, the contribution of variables was evaluated by the variable importance in the projection (VIP) values and the student’s *t*-test which was followed by the adjustment of the false discovery rate (FDR) by multiple hypothesis tests. The differential lipids were determined by a combination of FDR, fold change (FC) and VIP values, and a 10-fold cross-validation method was used to verify the accuracy of important lipids. Moreover, the correlation between the clinical parameters and lipid concentrations was analyzed by Pearson correlation. The potential biomarkers were evaluated by receiver operating characteristic (ROC) analysis after transformation by a logistic regression model to the predicted probability scale.

## Results

### Clinical Characteristics of the Recruited Participants

A total of 75 individuals were included in this study. No significant difference was found among the three groups in terms of age (*P* = 0.239) and BMI (*P* = 0.693). The scale score of HAMD, HAMA, and PANSS in MDD and BPD groups were higher than those of HC group (Table 1), and a significant difference in score of HAMA was also observed between MDD and BPD groups. Moreover, duloxetine and venlafaxine are the main therapeutic drugs used in MDD group and olanzapine or quetiapine combined with lithium or valproate are the main therapeutic drugs used in BPD group. It is worth noting that the recruited participants did not or occasionally drink alcohol and smoke. Detailed clinical and demographic characteristics of the studied samples are displayed in Supplementary Table 1.

**TABLE 1:** Comparison of clinical characteristics data and symptom scale assessment among the three groups.

Parameter	HC (*n* = 25)	MDD (*n* = 28)	BPD (*n* = 22)	*P*-value
Age [years, M(*P*_25_, *P*_75_)] *^b^*	31.00(26.50, 38.00)	36.50(30.25, 43.50)	34.00(27.00, 42.00)	0.239
BMI [kg/m^2^, mean ± SD (range)] *^a^*	20.93 ± 2.91 (16.98 – 26.73)	21.61 ± 3.00 (15.20 – 27.69)	21.57 ± 3.60 (14.68 – 27.99)	0.693
HAMD [M(*P*_25_, *P*_75_)] *^b^*	3.00(2.00, 5.50)	25.00(23.00, 26.75)*	20.00(9.25, 27.25)*	<0.001
HAMA [M(*P*_25_, *P*_75_)] *^b^*	4.00(2.50, 6.00)	25.50(24.00, 29.00)*	20.00(7.75, 23.25)*^#^	<0.001
PANSS [mean ± SD (range)] *^b^*	35.40 ± 3.61 (30.00 – 42.00)	62.04 ± 13.04 (40.00 – 90.00)*	61.55 ± 19.03 (31.00 – 98.00)*	<0.001

^a^one-way ANOVA; ^b^Kruskal Wallis; *P < 0.05 vs. HC group; ^#^< 0.05 vs. MDD group; BMI, body mass index; Values are shown as mean ± SD or M(P_25_, P_75_); SD, standard deviation.

### Alternation of Lipid at Class Level and Differential Carbon Chain Lengths and Degree of Saturation of Fatty Acyls in Major Depressive Disorder and Bipolar Depression

**FIGURE 1:**
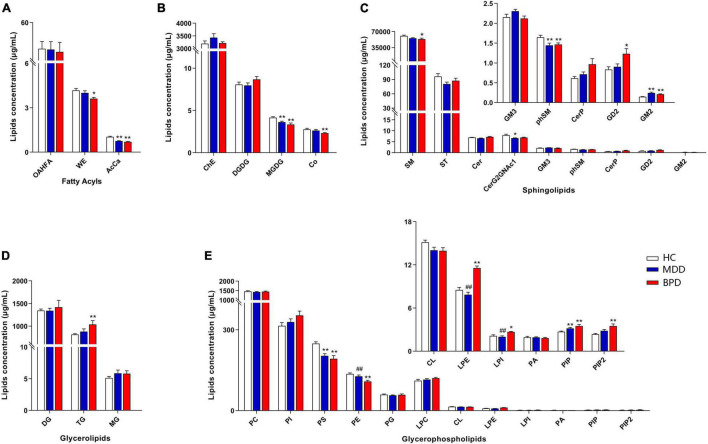
Differential concentration of lipid class among major depressive disorder (MDD), bipolar depression (BPD), and healthy controls (HC). **(A)** Fatty acyls, **(B)** ChE, Co, DGDG, and MGDG, **(C)** sphingolipids, **(D)** glycerolipids, **(E)** glycerophospholipids. HC, healthy controls; MDD, major depressive disorder; BPD, bipolar depression; OAHFA, (O-acyl)-1-hydroxy fatty acid; WE, wax esters; AcCa, acylcarnitine; ChE, cholesterol ester; Co, coenzyme; DGDG, digalactosyldiacylglycerol; MGDG, monogalactosyldiacylglycerol; SM, sphingomyelin; ST, sulfatide; Cer, ceramides; CerP, ceramides phosphate; phSM, phytosphingomyelin; DG, diglyceride; TG, triglyceride; MG, monoglyceride; PC, phosphatidylcholine; PI, phosphatidylinositol; PS, phosphatidylserine; PE, phosphatidylethanolamine; PG, phosphatidylglycerol; CL, cardiolipin; LPC, lysophosphatidylcholine; LPE, lysophosphatidylethanolamine; LPI, lysophosphatidylinositol; PA, phosphatidic acid; PIP, phosphatidylinositol; PIP2, phosphatidylinositol 4,5-bisphosphate. *P < 0.05 vs. HC; **P < 0.01 vs. HC, ^##^*P* < 0.01 vs. BPD.

A total of 31 lipid classes and 884 lipid species were identified in the samples of each group (Supplementary Table 2). Lipidomic analyses revealed that there are significant differences on the levels of several classes including wax esters (WE, *H* = 8.789, *P* = 0.012) and acylcarnitine (AcCa, *H* = 35.991, *P* < 0.001) (Figure 1A), monogalactosyldiacylglycerol (MGDG, *F* = 9.030, *P* < 0.001) and coenzymeQ10 (Co, *H* = 10.578, *P* = 0.005) (Figure 1B), sphingomyelin (SM, *H* = 9.671, *P* = 0.008), phytosphingomyelin (phSM, *F* = 4.995, *P* = 0.010), CeramideG2GNAc1 (Cer G2GNAc1 *H* = 8.795, *P* = 0.012) and GM2 (*H* = 25.646, *P* < 0.001) (Figure 1C), triglyceride (TG, *H* = 7.790, *P* = 0.020) (Figure 1D), phosphatidylserine (PS, *F* = 11.727, *P* < 0.001), phosphatidylethanolamine (PE, *F* = 9.885, *P* < 0.001), lysophosphatidylethanolamine (LPE, *F* = 33.952, *P* < 0.001), lysophosphatidylinositol (LPI, *H* = 13.570, *P* = 0.001), phosphatidylinositol (PIP, *H* = 15.034, *P* = 0.001) and phosphatidylinositol 4,5-bisphosphate (PIP2, *H* = 11.919, *P* = 0.003) (Figure 1E) among MDD, BPD and HC. Intercomparison further showed that levels of MGDG, phSM, CerG2GNAc1, AcCa and PS were decreased while GM2 and PIP were increased in MDD when compared with HC. Meanwhile, levels of MGDG, Co, SM, phSM, WE, AcCa, PS, and PE were decreased while GD2, GM2, TG, LPE, PIP, and PIP2 were increased in BPD when compared with HC. Moreover, levels of LPE and LPI were decreased while PE were increased in MDD when compared with BPD.

**FIGURE 2:**
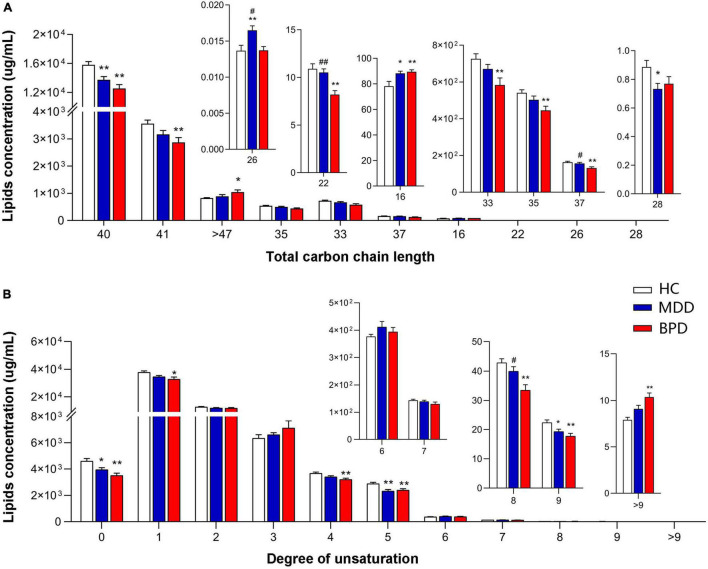
Groupwise alterations in fatty acid composition in the plasma. Results of analysis of fatty acyl composition by **(A)** chain length (number of carbons) and **(B)** degree of unsaturation. Lipids showing low levels results have been enlarged in insets. HC, health control; MDD, major depressive disorder; BPD, bipolar depression; *P < 0.05 vs. HC; **P < 0.01 vs. HC; ^#^*P* < 0.05 vs. BD; ^##^*P* < 0.01 vs. BPD.

To further investigate the difference in lipid composition, we compared the concentrations of lipids with different carbon chain lengths and the degree of saturation of the fatty acyls among MDD, BPD, and HC. As shown in Figure 2, we observed significant alterations in the fatty acyl chain profile of lipids in the plasma. Levels of long-chain fatty acyls with 40 carbons (40C) and levels of saturated fatty acid, polyunsaturated fatty acyls with 5 double bonds and 9 double bonds were decreased, while levels of long-chain fatty acyls with 16 carbons (16C) were increased in both the MDD (MDD vs. HC, *P* < 0.05) and the BPD group (BPD vs. HC, *P* < 0.05). Meanwhile, levels of long-chain fatty acyls with 26 carbons (26C) were increased, while level of long-chain fatty acyls with 28 carbons (28C) was decreased in the MDD group (MDD vs. HC, *P* < 0.05). Levels of long-chain fatty acyls with 41 carbons (41C), 37 carbons (37C), 35 carbons (35C), 33 carbons (33C) and polyunsaturated fatty acyls with 1 double bond, 4 double bonds and 8 double bonds were decreased, while levels of long-chain fatty acyls more 47 carbons (>47C) and polyunsaturated fatty acyls more than 9 double bonds were increased in the BPD group (BPD vs. HC, *P* < 0.05). Notably, a significant difference in the levels of long-chain fatty acyls with 26 carbons (26C), 22 carbons (22C), 37 carbons (37C), and polyunsaturated fatty acyls with 8 double bonds were also observed between the MDD and BPD group (MDD vs. BPD, *P* < 0.05). Taken together, there were obvious changes in lipid composition not only at lipid class level but also in the structure of fatty acyls among the three groups.

### Characteristic Lipid Species Between Major Depressive Disorder and Healthy Controls

**FIGURE 3:**
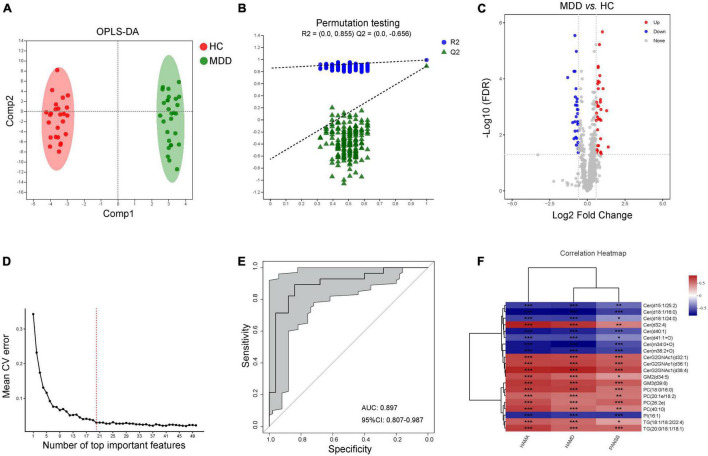
Characteristic lipid species in MDD. **(A)** Scatter plot of OPLS-DA model and **(B)** validation model of permutation test, **(C)** volcano map reveals the increased (red dots) and decreased (blue dots) lipid species between the MDD and HC, **(D)** random forest model and 10-fold cross-validation showed that the lipids with the top 20 importance had the lowest model error rate, **(E)** ROC analysis for the combinational lipids, **(F)** correlation between clinical parameters and levels of 20 lipid species. *P < 0.05; **P < 0.01, ***P < 0.001.

According to the characteristics of the OPLS-DA model, the data of plasma lipids could be well distinguished between HC and MDD (Figure 3A). The OPLS-DA model was reliable to screen lipid biomarkers between HC and MDD (Figure 3B). The differential lipids were determined based on the following criteria: (1) FC > 1.5 or < 0.067, (2) VIP > 1, and (3) FDR value < 0.05. A total of 66 differential lipid species were identified (36 up-regulated and 30 down-regulated in MDD) between MDD and HC (Figure 3C and Supplementary Table 3). Meanwhile, the above molecules were selected to construct a random forest model, and 10-fold cross-validation showed that the lipids with the top 20 importance had the lowest model error rate (Figure 3D). These 20 lipids covered 7 different lipid class and showed good sensitivity and specificity [AUC = 0.897, 95% CI (0.807, 0.978)] (Figure 3E). Moreover, among these characteristic species, levels of Cer(d15:1/25:2), Cer(d18:1/16:0), Cer(d18:1/24:0), Cer(d40:1), Cer(d41:1 + O), Cer(m34:0 + O), Cer(m38:2 + O) and PI(16:1) were negatively while levels of Cer(d32:4), CerG2GNAc1(d32:1), CerG2GNAc1(d36:1), CerG2GNAc1(d38:4), GM2(d34:5), GM3(t39:6), PC(18:0/16:0), PC(20:1e/18:2), PC(26:2e), PC(40:10), TG(18:1/18:2/22:4) and TG(20:0/18:1/18:1) were positively correlated with the scores of HAMA, HAMD and PANSS (Figure 3F), indicating those lipids in the plasma might be a combinational biomarker for women with MDD.

### Characteristic Lipid Species Between Bipolar Depression and Healthy Controls

**FIGURE 4:**
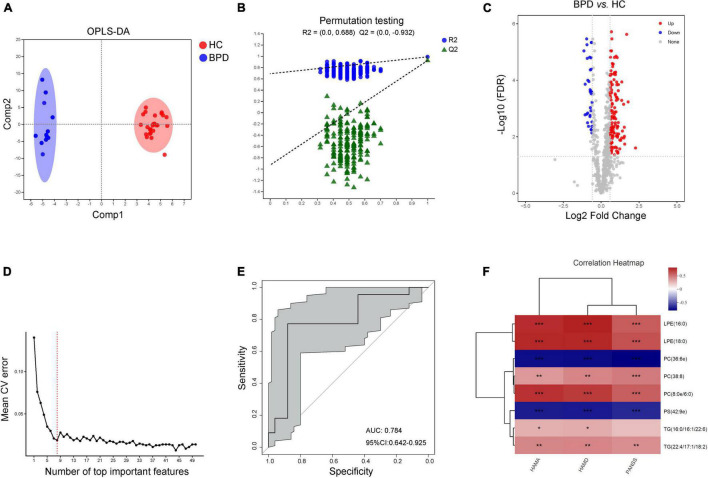
Characteristic lipid species in BPD. **(A)** Scatter plot of OPLS-DA model and **(B)** validation model of permutation test, **(C)** volcano map reveals the increased (red dots) and decreased (blue dots) lipid species between the BPD and HC, **(D)** random forest model and 10-fold cross-validation showed that the lipids with the top 8 importance had the lowest model error rate, **(E)** ROC analysis for the combinational lipids, **(F)** correlation between clinical parameters and levels of 8 lipid species. *P < 0.05; **P < 0.01, ***P < 0.001.

Data of plasma lipids could also be well distinguished and reliable to screen lipid biomarkers between HC and BPD (Figures 4A,B). A total of 172 differential lipid species were identified (138 up-regulated and 34 down-regulated in BPD) between BPD and HC (Figure 4C and Supplementary Table 4). 10-fold cross-validation further showed that the lipids with the top 8 importance had the lowest model error rate (Figure 4D). These 8 lipids covered 4 different lipid class and showed good sensitivity and specificity [AUC = 0.784, 95% CI (0.642, 0.925)] (Figure 4E). Moreover, among these characteristic species, levels of PC(36:6e) and PS(42:9e) were negative while levels of LPE(16:0), LPE(18:0), PC(38:8), PC(8:0e/6:0) and TG(22:4/17:1/18:2) were positively correlated with the scores of HAMA, HAMD and PANSS (Figure 4F). Meanwhile, levels of TG(16:0/16:1/22:6) were also positively correlated with the scores of HAMA and HAMD. Taken together, these 8 lipids might be a combinational plasma biomarker for women with BPD.

### Characteristic Lipid Species Between Major Depressive Disorder and Bipolar Depression

**FIGURE 5:**
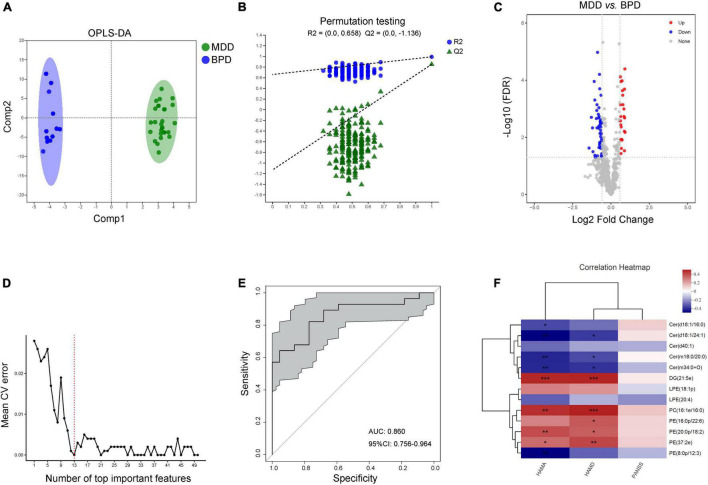
Characteristic lipid species between MDD and BPD. **(A)** Scatter plot of OPLS-DA model and **(B)** validation model of permutation test, **(C)** volcano map reveals the increased (red dots) and decreased (blue dots) lipid species between the MDD and BPD, **(D)** random forest model and 10-fold cross-validation showed that the lipids with the top 13 importance had the lowest model error rate, **(E)** ROC analysis for the combinational lipids, **(F)** correlation between clinical parameters and levels of 13 lipid species. *P < 0.05; **P < 0.01, ***P < 0.001.

Moreover, data of plasma lipids could be well distinguished and reliable to screen lipid biomarkers between MDD and BPD as well (Figures 5A,B). A total of 77 differential lipid species were identified (25 up-regulated and 52 down-regulated in MDD) between MDD and BPD (Figure 5C and Supplementary Table 5). Meanwhile, 10-fold cross-validation showed that the lipids with the top 13 importance had the lowest model error rate (Figure 5D). These 13 lipids covered 5 different lipid class and showed good sensitivity and specificity [AUC = 0.860, 95% CI (0.756, 0.964)] (Figure 5E). Moreover, among these characteristic species, levels of Cer(d18:1/24:1), Cer(m18:0/20:0) and Cer(m34:0 + O) were negatively while levels of DG(21:5e), PC(16:1e/16:0), PE(20:0p/18:2) and PE(37:2e) were positively correlated with the scores of HAMA and HAMD (Figure 5F). Meanwhile, levels of Cer(d18:1/16:0) and PE(8:0p/12:3) were negatively correlated with the scores of HAMA and levels of PE(16:0p/22:6) were positively correlated with the scores of HAMD. However, there was no difference in correlation analysis between levels of these species and scores of PANSS. Thus, there are differences in lipidomics between women with depression and bipolar depression, and these 13 lipids might be a potential combinational plasma biomarker to distinguish the two diseases.

## Discussion

In this study, we characterized the peripheral lipid composition of adult women with MDD and BPD and identified unique lipids signatures of subjects with MDD and BPD relative to HC. Meanwhile, we identified diagnostic potential biomarkers comprising several lipids, which may distinguish MDD from BPD, and each from HC, with moderate reliability. Although the result is worth further exploration in clinical trials with large sample size, these results may help identify novel clinical diagnostic and therapeutic targets for adult women with MDD and BPD.

Changes in lipid composition have been reported in several neuropsychiatric diseases (45, 46), such as MDD and BD (47, 48). However, thus far, the potential lipids that can distinguish women with MDD from BPD have not been identified. Such investigations would be particularly valuable to understand the shared and distinct lipid characteristics between these two disorders. Herein, we found that the levels of MGDG, phSM, AcCa and PS were decreased while GM2 and PIP were increased both in MDD and BPD when compared with HC. Supporting this finding, previous studies found that plasma SM was negatively correlated with depressive symptoms (49) and lower concentrations of AcCa were observed in the plasma or serum in MDD and BD patients when compared with healthy control previously (50–52). Moreover, alterations in AcCa were also involved in the action of citalopram/escitalopram in major depression (53). On the other hand, although there is no direct evidence, MGDG shows a strong anti-inflammatory property (54, 55) and PS acts as an engulfment signal for phagocytosis in apoptotic cells and participates in immune regulation in non-apoptotic cells (56, 57). Meanwhile, disrupted GM2/GD2 synthase was associated with neurodegeneration disease (58) and GM2 elevation was even related to glial activation induced damage in the developing brain (59). In addition, PI and its array of inositol-phosphorylated products PIP and PIP2, play fundamental roles in the regulation of membrane trafficking and cytoskeletal dynamics as well as cell biology that include membrane-delineated signal transduction, as well as (60, 61), and serum PI might be a biomarker for bipolar disorder (62). These results suggest that there are some similar lipid changes in depression and BPD, and these lipids are related to energy metabolism and oxidative stress, inflammation and neurodegenerative diseases (63, 64). Notably, levels of LPE and LPI were decreased while PE was increased in MDD when compared with BPD. Given that lysophospholipids inhibited *N*-methyl-D-aspartate (NMDA) responses (65) whereas PE influenced events for the remission of acute inflammation and energy metabolism (66, 67), this difference may be related to the peripheral inflammatory response and excitability regulation between the unipolar and bipolar depression.

It should be noted that the changed lipids between the BPD and HC were more obvious than that of MDD vs. HC. Previous works found that PE was decreased whereas TG was elevated in the plasma of BD (68, 69). Due to PS biosynthesis *via* the serine base-exchange reaction using PE (70), the decrease in PS levels may be related to the reduction of PE. Meanwhile, LPE and PI showed pronounced positive relationships with depression severity (71). Similarly, CoQ10 might be considered as an effective strategy for the treatment of bipolar depression (72) whereas lithium significantly decreased PIP2 levels in the platelet membrane of BD (73, 74). Likewise, here we found that levels of CoQ10, SM, PS, and PE were decreased while TG, LPE, PIP, and PIP2 were increased in BPD when compared with HC. It has been reported that plasma Cer levels increase in patients with major depression and bipolar disorder (39) and the Cer might be a novel potential antidepressant target (75, 76). Unfortunately, we did not observe a difference in Cer levels between MDD and HC, as well as did in BPD vs. HC. This inconsistency may be related to the gender, age and dietary structure of the subjects included in this study. Of course, the interference of drugs cannot be ruled out. Furthermore, as the carbon chain length and the degree of saturation of the fatty acids influence biophysical properties of lipids (77, 78), the present study also observed the alterations in the concentrations of different carbon chains and unsaturated fatty acids in the plasma of each group. Although the potential mechanism for this difference is not clear, this result may further explain the difference in lipid molecular structure and function in female MDD and BPD.

In terms of lipid species, data of plasma lipids could be distinguished into the three groups (Supplementary Figure 1A). By the trends of changes in lipids class, a total of 66 differential lipid species were identified between MDD and HC whereas 172 differential lipid species were identified between BPD and HC. Meanwhile, 77 differential lipid species were identified between MDD and BPD. Coverage of lipids in all three comparisons showed that there are 20 unique lipids found in MDD vs. HC and 28 unique lipids for MDD vs. BPD (Supplementary Figure 1A and Supplementary Table 6). However, only 37 differentially regulated lipids were identified for MDD in drug-free patients and 121 statistically differential lipids between the BPD and HC in serum lipidomic analysis in previous studies (37, 38). Therefore, the present study newly reports some previously unidentified lipid signatures associated with female MDD or BPD and lays a foundation from which to further characterize the distinct plasma lipids underpinnings of MDD and BPD. Besides identifying the lipid compositions that characterized each disease, the present study also identified a signature of 20 lipids that could distinguish patients with MDD from HC, with an AUC value of 0.897 and a signature of 8 lipids that could distinguish patients with BPD from HC, with an AUC value of 0.784. Notably, a signature of 13 lipids was identified that could distinguish MDD from BPD, with an AUC value of 0.860. We believe this method has potential as a tool for distinguishing MDD from BPD (and both from HC) based on subjects’ plasma lipidomics. However, the identified potential lipid biomarkers were also inconsistent with previous works (71, 79), this discrepancy may be related to gender and symptom characteristics, sample type and analysis method which needs to be verified by larger samples in the future.

The dysfunction of lipid metabolism in peripheral blood may contribute to the etiopathology of MDD and BD (79, 80), and the use of other treatments aimed at modulating the function and regulation of the lipids such as phospholipid and sphingolipid represent a means to improve or prevention in individuals suffering from psychiatric disorders (36, 81, 82). However, the crosstalk between brain and peripheral lipid homeostasis is still largely unknown. Analysis of lipids and associated enzymes in cerebrospinal fluid might be provided further insight to explain the relationship between central lipid homeostasis and symptom characteristics of MDD and BPD. Furthermore, the most probable period of the first episode of MDD extends from mid-adolescence to mid-40s (83). In comparison, the prevalence of BD was evident from 10 years of age and peaked in the early 20s (84). Although the average age range of patients included in this study was 30–40 years old, the influence of age factors on lipid composition is partly excluded. In particular, menopause is likely to affect lipid metabolism (85, 86) and there were 9 cases over 45 years old in this study, the effect of menopause on plasma lipidomics needs to be further considered. On the other hand, other signatures that can discriminate unipolar from bipolar depression including gut microbiota, imaging features and metabolomics were reported recently (87–89), the advantages, disadvantages and interaction between lipidomic characteristics and those signatures still need to be further explored. Finally, the potential limitations of the study should be mentioned. (i) The number of recruited samples is relatively small; (ii) environmental or regional-specific biases on subjects’ lipids compositions cannot be ignored; (iii) the influence of drugs on lipid composition cannot be excluded. It is well known that antipsychotic drugs could affect lipid metabolism and olanzapine and quetiapine were used in the present study (90); (iv) our findings cannot show a causal relationship between the identified differential lipid compositions in BPD or MDD. A discovery and validation cohort research with larger sample sizes should be operated to verify the potential use of these lipid markers and it may be useful to further identify the potential biomarkers which could reflect the differences with the duration of disease or age group in the future.

## Conclusion

In summary, the present study characterized and identified different plasma lipids compositions in adult women with MDD vs. BPD, and in both vs. HC. Furthermore, lipid classifiers that could be able to discriminate MDD from BPD and HC were developed. Our findings lay the potential foundation for further development of plasma lipidomic-based clinical diagnostic assay for MDD and BPD.

## Data Availability Statement

The original contributions presented in this study are included in the article/Supplementary Material, further inquiries can be directed to the corresponding authors.

## Ethics Statement

The study involving humans was reviewed and approved by the Ethics Committee of Chang’an Hospital (approval number: ChANLL2020001). The patients/participants provided their written informed consent to participate in this study.

## Author Contributions

TZ, LG, Z-WP, and Q-RT designed the experiment protocol and responsible for the data collection. RL, FW, and W-MY were responsible for the clinical evaluation. J-BY and Z-QC were responsible for the data management. C-HZ, Y-HC, and HY analyzed the data. TZ and LG wrote the first draft. Z-WP and Q-RT financed the study and revised the manuscript. All authors contributed to the article and approved the submitted version.

## Conflict of Interest

The authors declare that the research was conducted in the absence of any commercial or financial relationships that could be construed as a potential conflict of interest.

## Publisher’s Note

All claims expressed in this article are solely those of the authors and do not necessarily represent those of their affiliated organizations, or those of the publisher, the editors and the reviewers. Any product that may be evaluated in this article, or claim that may be made by its manufacturer, is not guaranteed or endorsed by the publisher.

## Supplementary Material

Supplementary Figure 1 **(A)** Scatter plot of OPLS-DA model for three groups and **(B)** coverage of lipids in all three comparisons.
