# Supplementary figures and images for: Alterations of Plasma Lipids in Adult Women With Major Depressive Disorder and Bipolar Depression
Source: Front Psychiatry. 2022 Jul 18;13:927817. doi: 10.3389/fpsyt.2022.927817 (PMC9339614; doi:10.3389/fpsyt.2022.927817)

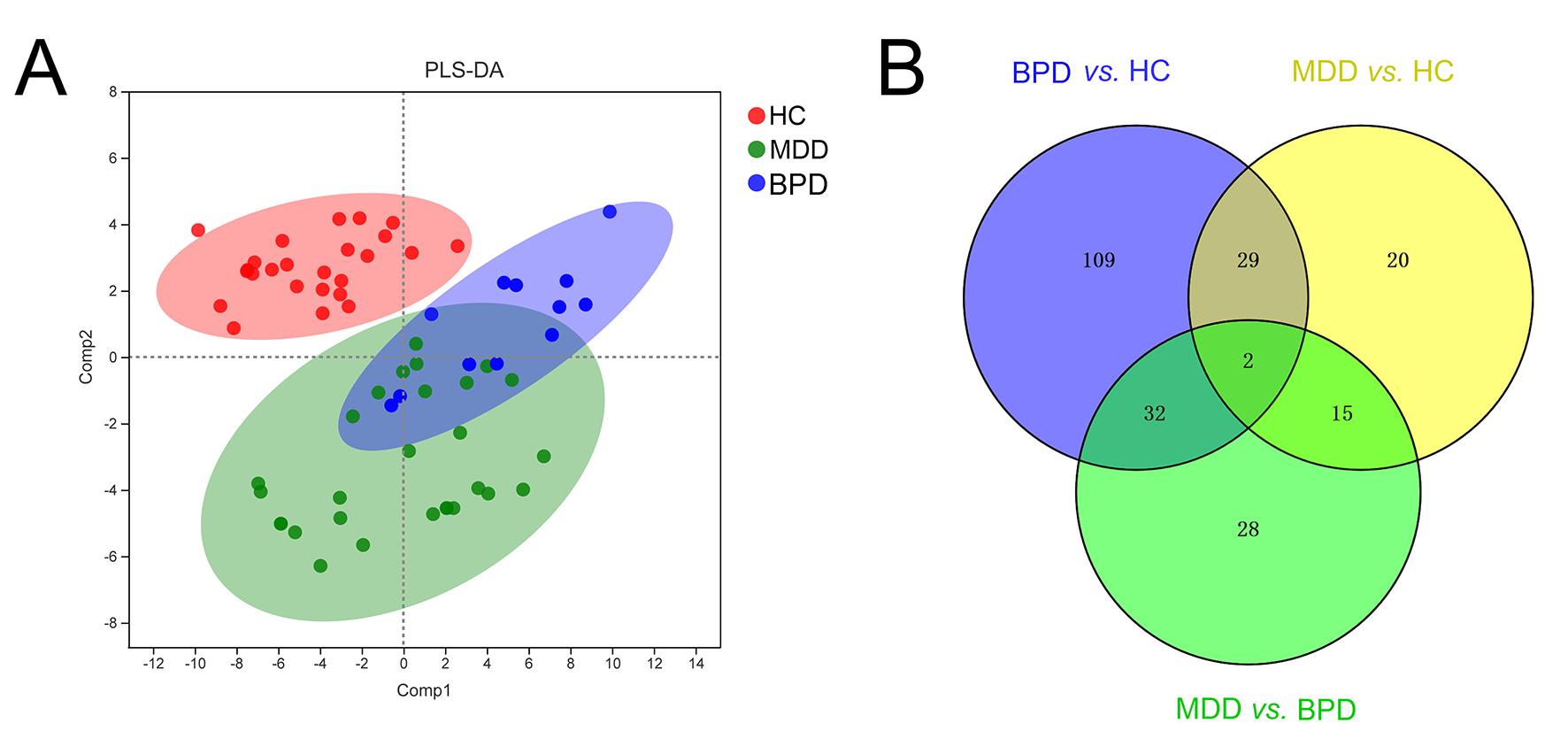

Supplement: Supplementary Figure 1 — (A) Scatter plot of OPLS-DA model for three groups and (B) coverage of lipids in all three comparisons. [file Image_1.TIF]
